# Jamestown Canyon Virus in Collected Mosquitoes, Maine, United States, 2017–2019

**DOI:** 10.3201/eid2811.212382

**Published:** 2022-11

**Authors:** Elizabeth F. Schneider, Rebecca M. Robich, Susan P. Elias, Charles B. Lubelczyk, Danielle S. Cosenza, Robert P. Smith

**Affiliations:** MaineHealth Institute for Research, Scarborough, Maine, USA

**Keywords:** Jamestown Canyon virus, viruses, vector-borne infections, zoonoses, orthobunyavirus, mosquitoes, phylogenetics, Maine, United States

## Abstract

Jamestown Canyon virus (JCV) is a mosquito-borne arbovirus that circulates in North America. We detected JCV in 4 pools of mosquitoes collected from midcoastal Maine, USA, during 2017–2019. Phylogenetic analysis of a JCV sequence obtained from *Aedes cantator* mosquitoes clustered within clade A, which also circulates in Connecticut, USA.

Jamestown Canyon virus (JCV; family *Peribunyaviridae*, genus *Orthobunyavirus*) is a mosquitoborne virus that belongs to the California serogroup. Although rare, JCV infection in humans can cause acute febrile encephalitis, meningitis, and meningoencephalitis (*1*). JCV was identified from *Culiseta inornata* mosquitoes in Jamestown Canyon, Colorado, USA, in 1961 (*2*). Since then, JCV has been detected in humans in the United States and Canada (*1*). 

JCV has been isolated from >26 species of mosquitoes belonging to *Aedes/Ochlerotatus*, *Anopheles*, *Coquillettidia*, *Culex*, *Culiseta*, and *Psorophora* genera (*3*,*4*). White-tailed deer (*Odocoileus virginianus*) are likely the primary amplifying host of JCV (*5*), but moose (*Alces alces*), elk (*Cervus elaphus*), and bison (*Bison bison*) also might contribute to the transmission cycle (*6*). In Maine, moose and white-tailed deer are distributed statewide (*7*).

In 2017, two confirmed symptomatic human JCV cases were reported in Maine, and a subsequent fatal case was reported in the state in 2018 (*8*). All 3 cases occurred in women >65 years of age who resided in 3 counties: Kennebec, Franklin, and Knox (Figure 1) (*8*). Because JCV was recently identified in Maine, mosquito testing could help delineate the geographic distribution of JCV in the state. We collected and tested mosquitoes for JCV to obtain viral genomic sequences, conduct phylogenetic comparison, and determine whether JCV from Maine was congruent with published JCV sequences from the northeastern United States.

**Figure 1:**
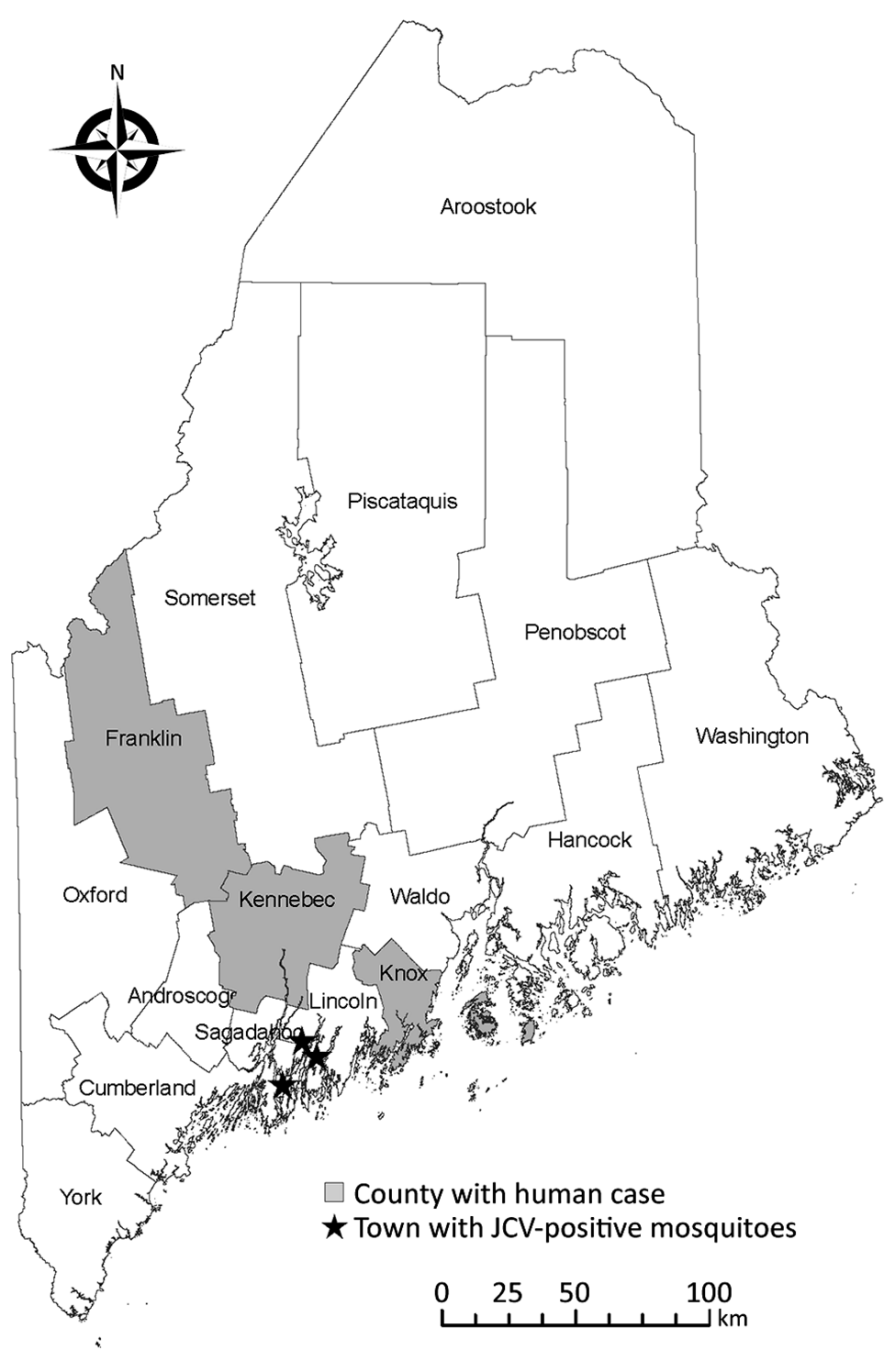
Locations of JCV in humans and collected mosquitoes, Maine, USA, 2017–2019. JCV-positive mosquitoes were found in the town of Arrowsic in Sagadahoc County and in the towns of Edgecomb and Wiscasset in Lincoln County during 2017–2019. In 2017, two confirmed symptomatic human JCV cases were reported; a third fatal human case was reported in 2018. JCV, Jamestown Canyon virus.

## The Study

We trapped mosquitoes during mid-June–September each year during 2017–2019 in 36 towns in 9 of Maine’s 16 counties, representing southern, midcoastal, and northern regions of the state. We used CDC Miniature Light Traps (John W. Hock Co., https://www.johnwhock.com) baited with CO_2_ by using dry ice. We deployed 1 trap per site once per week and set the traps to run overnight from ≈2:00 PM–10:00 AM Eastern Standard Time. We identified mosquitoes’ sex and species and pooled only female mosquitoes by species, collection site, and collection date, <50 mosquitoes per pool.

We extracted RNA from mosquito pools by using the QIAmp Viral RNA Mini Kit (QIAGEN, https://www.qiagen.com) following manufacturer protocol. We tested pools for JCV by reverse transcription PCR (RT-PCR) by using the SuperScript III One-Step RT-PCR System with Platinum Taq DNA polymerase (Invitrogen, https://ww.invitrogen.com) and primers designed to amplify 24 viruses within the Bunyamwera-California complex, including JCV (*9*).

We subsequently analyzed mosquito pools that tested positive for JCV RNA by using JCV-specific primers that target a 605-bp region of the nucleocapsid and nonstructural genes within the small segment (*9*). We conducted RT-PCR in the same manner described above but used Platinum *Taq* High Fidelity DNA Polymerase (Invitrogen). The University of Maine DNA Sequencing Facility (Orono, ME, USA) sequenced positive samples obtained from both primer sets. We confirmed JCV identities by BLASTn (https://blast.ncbi.nlm.nih.gov/Blast.cgi).

We compared 1 positive sequence against 18 previously published orthobunyaviruses obtained from GenBank. We performed phylogenetic analysis in MEGA X (https://www.megasoftware.net) by using the neighbor-joining method and maximum composite likelihood model. We calculated 1,000 bootstrap replicates to provide support for each node.

## Conclusions

During 2017–2019, we collected 13,023 mosquitoes from 36 towns in 9 counties in Maine, a total of 162 trap nights. We tested a total of 689 mosquito pools representing 24 species for the presence of JCV RNA by RT-PCR. Among all pools, 4 (0.6%) pools representing 4 (16.6%) different species were positive for JCV viral RNA (Table 1).

**Table 1:** Summary of female mosquitoes tested by reverse transcription PCR for Jamestown Canyon virus, Maine, USA, 2017–2019*

Mosquito species	No. pools	Total no. mosquitoes	JCV-positive pools†
*Aedes abserratus/punctor*	31	439	0
*Ae. canadensis*	104	1,724	0
*Ae. cinereus*	7	31	0
*Ae. cantator*	70	1,773	1
*Ae. excrucians*	24	391	0
*Ae. fitchii*	1	2	0
*Ae. hendersoni*	9	65	0
*Ae. intrudens*	4	18	0
*Ae. japonicus*	12	48	0
*Ae. provocans*	46	426	1
*Ae. sollicitans*	12	116	1
*Ae.* species	2	77	0
*Ae. sticticus*	1	3	0
*Ae. stimulans*	4	29	0
*Ae. taeniorhynchus*	1	2	0
*Ae. triseriatus*	28	183	0
*Ae. vexans*	24	198	0
*Anopheles punctipennis*	79	271	0
*An. quadrimaculatus*	17	65	0
*An. walkeri*	1	3	0
*Coquillettidia perturbans*	200	7,081	0
*Culiseta melanura*	2	4	0
*Culex pipens/restuans*	6	63	0
*Cx. salinarius*	1	5	0
*Uranotaenia sapphirina*	3	6	1
Total	689	13,023	4

*JCV, Jamestown Canyon virus. †Pools include <50 female mosquitoes/pool.

We detected JCV RNA in each of the 3 years of the study: in 1 positive pool of *Aedes provocans* mosquitoes in 2017; 2 positive pools in 2018, 1 each of *Ae. sollicitans* and *Uranotaenia* *sapphirina* mosquitoes; and 1 positive pool of *Ae. cantator* mosquitoes in 2019. All sequences matched other JCV sequences in GenBank with >99% identity. All JCV-positive mosquito pools were collected during a 3-week period, June 30–July 19. Although the testing effort represented the southern, midcoastal, and northern parts of the state, the positive mosquito pools originated from 3 towns in 2 midcoastal counties, Arrowsic in Sagadahoc County and Edgecomb and Wiscasset in Lincoln County (Figure 1).

**Figure 2:**
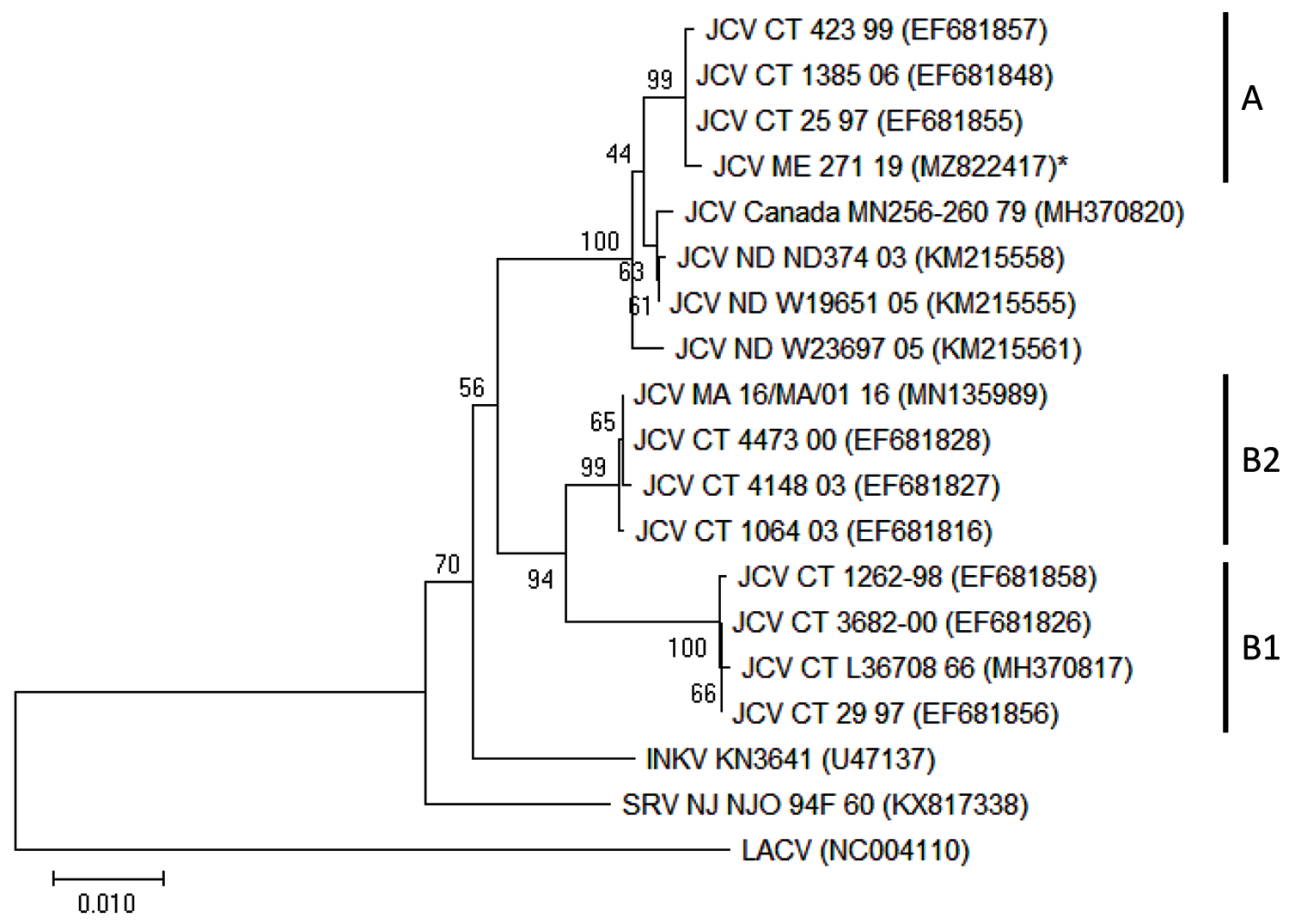
Phylogenetic analysis of JCV from collected mosquitoes, Maine, USA, 2017–2019. We compared a JCV sequence detected in mosquitoes from Maine to sequences from JCV and other viruses detected in other areas of the United States and Canada. We analyzed sequences by using the neighbor-joining method in MEGA X (https://www.megasoftware.net). The state or region of origin, strain, and year of isolation or detection are indicated for each virus, when available; GenBank accession numbers are provided. Asterisk indicates the sequence generated in this study. Numbers at branch nodes represent bootstrap values. Virus clades are indicated on the right. Scale bar indicates nucleotide substitutions per site. INKV, Inkoo virus; JCV, Jamestown Canyon virus; LACV, La Crosse virus; SRV, South River virus.

Because of a storage freezer failure, we were only able to resequence 1 of the original 4 JCV-positive pools with the second set of primers. We chose this sequence for phylogenetic analysis because it provided us with a larger portion of the genome and would be more robust for analysis. This JCV-positive pool was from *Ae. cantator* mosquitoes collected in the town of Edgecomb, Lincoln County, in July 2019. Phylogenetic analysis of the Edgecomb sequence (GenBank accession no. MZ822417) and 18 other sequences obtained from GenBank showed this JCV-positive sequence clustered within clade A described by a previous study (*10*), and had 99% nucleotide identity match with a JCV isolate from Connecticut collected in 2004 (GenBank accession no. HM007356) (Figure 2).

We detected JCV-positive mosquitoes in Maine, including 1 pool of *Ur. sapphirina* mosquitoes, a species not known as a JCV vector. In the southeastern United States, the *Ur. sapphirina* mosquito is considered a specialist of amphibians (*11*) and annelids (ringed worms or segmented worms), and 1 study from Florida found 100% of bloodmeals taken by *Ur. sapphirina* mosquitoes were from annelid hosts (*12*). However, in the northeastern United States, *Ur. sapphirina* mosquitoes appear to be generalists. In Connecticut, white-tailed deer have been identified as the most common vertebrate host for *Ur. sapphirina* mosquitoes, but additional bloodmeals from humans, birds, and reptiles are reported (*13*). The opportunistic feeding pattern of *Ur. sapphirina* mosquitoes in the northeast suggests this species might play a role in regional virus transmission.

In addition to *Ur. sapphirina* mosquitoes, we detected JCV RNA in *Ae. cantator*, *Ae. provocans*, and *Ae. sollicitans* mosquitoes, species known as mammalian pests that readily bite humans (*14*). The *Ae. provocans* mosquito is a known vector of JCV in New York, USA (*15*), and might serve as an overwintering reservoir (*4*). In Connecticut, *Ae. cantator* and *Ae. sollicitans* mosquito populations peak during late May through June and breed in saltmarshes and brackish water, which are common habitats along midcoastal Maine (*14*). *Ae. canadensis* mosquitoes have been identified as a dominant JCV vector in Connecticut (*4*). Although *Ae. canadensis* and *Coquillettidia perturbans* mosquitoes comprised most (44%) pools in our study, we did not detect JCV RNA in either species.

All JCV-positive mosquito pools in our study came from coastal counties, whereas the 3 human JCV cases during our study period came from 2 inland counties and 1 coastal county. Our sampling and testing effort was greater in the midcoastal region than in other regions of the state. A serosurvey for JCV antibodies in deer and moose in Maine might show a broader geographic extent than mosquito positivity and human cases (*7*).

In conclusion, the JVC sequence we obtained from *Ae. cantator* mosquitoes collected in 2019 from Edgecomb, in Lincoln County, Maine, clustered within clade A described by a previous study in Connecticut (*10*), where clade A is the most common clade, in addition to clades B1 and B2. Increased mosquito collection, testing effort, and phylogenetic analysis could elucidate the roles of particular mosquito species in JCV transmission, and better delineate the statewide phylogeographic distribution of JCV in Maine. Clarifying the distribution of JCV in mosquitoes in Maine can inform prevention efforts in the state. 
